# High genomic diversity of novel phages infecting the plant pathogen *Ralstonia solanacearum*, isolated in Mauritius and Reunion islands

**DOI:** 10.1038/s41598-021-84305-7

**Published:** 2021-03-08

**Authors:** Angélina Trotereau, Claudine Boyer, Isabelle Bornard, Max Jean Bernard Pécheur, Catherine Schouler, Clara Torres-Barceló

**Affiliations:** 1 INRAE, ISP, Université de Tours, 37380 Nouzilly, France; 2 Plant Populations and Bio-aggressors in Tropical Ecosystems, Saint Pierre, Reunion France; 3 Plant Pathology, INRAE, 84140 Montfavet, France; 4 Plant Pathology Division, FAREI, Reduit, Mauritius

**Keywords:** Genetics, Microbiology

## Abstract

Bacterial wilt caused by the *Ralstonia solanacearum* species complex (RSSC) is among the most important plant diseases worldwide, severely affecting a high number of crops and ornamental plants in tropical regions. Only a limited number of phages infecting *R. solanacearum* have been isolated over the years, despite the importance of this bacterium and the associated plant disease. The antibacterial effect or morphological traits of these *R. solanacearum* viruses have been well studied, but not their genomic features, which need deeper consideration. This study reports the full genome of 23 new phages infecting RSSC isolated from agricultural samples collected in Mauritius and Reunion islands, particularly affected by this plant bacterial pathogen and considered biodiversity hotspots in the Southwest Indian Ocean. The complete genomic information and phylogenetic classification is provided, revealing high genetic diversity between them and weak similarities with previous related phages. The results support our proposal of 13 new species and seven new genera of *R. solanacearum* phages. Our findings highlight the wide prevalence of phages of RSSC in infected agricultural settings and the underlying genetic diversity. Discoveries of this kind lead more insight into the diversity of phages in general and to optimizing their use as biocontrol agents of bacterial diseases of plants in agriculture.

## Introduction

Bacteria from the *Ralstonia solanacearum* species complex (RSSC) are major plant pathogens, causing bacterial wilt disease in a wide range of important agricultural and wild plant hosts^1^. Its distribution is worldwide, with special incidence in tropical areas, and detailed epidemiological information is available from many regions^2,3^. It is considered a bacterial species complex that comprises three species (*R. pseudosolanacearum*, *R. solanacearum* and *R. syzygii*), four genetic groups or phylotypes (I, II, III and IV) and more than 50 monophyletic groups, named sequevars^4^. *Ralstonia solanacearum* is included in the A2 (high risk) list of quarantine organisms in Europe and Canada, and some sequevars are listed as bioterrorist agents in the US, because of their potential danger to agriculture. Climate change and international commercial exchanges are increasing the risk of bacterial wilt outbreaks in presently unaffected areas^5,6^.

Bacteriophages or phages, the viruses of bacteria, are present in all environments and are an important ecological and evolutionary force that shape microbial communities^7^. It has been demonstrated that *R. solanacearum* phages can influence plant health or disease. Virulent phages can be used as biocontrol agents to limit the incidence of the disease, as shown recently^8^. Then, a particular group of phages, the non-lethal and filamentous *Inoviridae*, can either increase or decrease bacterial virulence, by providing with new genetic repertoire or modifying behaviours such as motility or biofilm formation in bacteria^9,10^. Several *R. solanacearum* phages have been studied, but the understanding of their prevalence in nature, or their complete characterisation is still poor.

The complete genome of only 35 *R. solanacearum* phages are fully classified according to the International Committee on Taxonomy of Viruses (ICTV) guides. This number is low compared, for example, to the 224 phages of *Pseudomonas* (comprising all bacterial species), or phages infecting human associated bacteria such as the 270 genomes of *Escherichia* phages. The amount of *R. solanacearum* phages described is however comparable to phages of the other five main plant pathogenic bacteria with worldwide distribution, with 45 *Erwinia*, 35 *Pectobacterium*, 21 *Rhizobium*, 18 *Xanthomonas* or 5 *Xylella* complete phage genomes published so far. The main geographical origin of the published complete *R. solanacearum* phage genomes is Asia, with 64% of all phages coming from Japan (e.g.^11^). There are no *R. solanacearum* phages from European sources among those fully-classified *R. solanacearum* phage genomes, even if recent work has isolated and tested phages as biocontrol agents of RSSC^12^. Only one phage genome is available from Africa^13^, despite that the agriculture of this continent suffers from bacterial wilt disease, and RSSC are present in most tropical areas. Despite the difficulties of analysis on non-conserved highly recombinant phage genomes, to obtain well-annotated genomes of virulent phages is essential for phage applied purposes, ensuring that they do not encode genes associated with toxicity in bacteria, or allowing to assess better the outcome of the treatment^14^. More genomic and metagenomic data of phages is helping to acquire detailed knowledge on phage ecology and evolution in varied ecosystems^15^.

New methods of phage genome analysis give the opportunity to undertake deep genetic characterisation of all phages and to do comparative genomics^16,17^. The ICTV has recently re-organized the taxonomy of phages, providing with guide-lines to classify phages^18^. The GenBank database includes the updated taxonomy of the 35 *R. solanacearum* phages. Thirteen are classified as *Autographiviridae*, eleven as *Inoviridae*, seven as *Myoviridae*, two as *Ackermanviridae* and only one as *Siphoviridae* and one as *Podoviridae*. Regarding the non Inoviridae, the *Ackermanviridae, Siphoviridae* and *Podoviridae* have not been assigned to genera. Of the remaining 20 *Autographiviridae* and *Myoviridae*, they are all comprised in 14 genera. The current study examined *R. solanacearum* phages from two African islands in the Southwest Indian Ocean, considered biodiversity hotspots: Mauritius and Reunion. The aim was to isolate and characterize *R. solanacearum* phages from a non-explored geographical region and thus to contribute knowledge on viral diversity. We report the discovery of 23 new *R. solanacearum* phages, and propose 13 new species and 7 new genera, based on genomic and morphological analyses. We also complete the taxonomic classification of three *R. solanacearum* phages already described but not fully characterised. We highlight the genetic diversity of these newly isolated phages that contributes considerably to expanding our understanding of *R. solanacearum* phages.

## Methods

### Sampling, isolation and purification of phages

Sampling of *R. solanacearum* phages in this study was carried out at four agricultural sites in Southwestern Reunion Island in February 2018 and at six sites in Mauritius Island in March 2018 (see exact locations and maps in Supplementary Fig. 1). The criteria for site selection was the recent incidence of bacterial wilt disease in solanaceous plants, irrespective of location, host plant species or cultivation method (greenhouse, field, off-ground, organic, etc.). Different types of samples were collected: diseased plant roots, soil and irrigation water. The locations and characteristics of the samples from each of the sites with positive samples are presented in Supplementary Table 1.

An enrichment technique with multiple bacterial strains was used to widen the diversity of phage isolation^19^. We used the most frequent *R. pseudosolanacearum* strains founded in each island as well as other strains that represent the genetic diversity of RSSC (Supplementary Table 2). The strains were not mixed in the same tube due to the possible production of bacteriocins by RSSC^20^. Each agricultural sample was divided and enriched with every strain in separate tubes. Using the overlay assay, single plaques were identified and transferred three times. A RSSC strain from sequevar I-31, the main genetic variant found in Reunion, was used as the common host for subsequent amplification of Reunion Island phage clones^3^. Each phage clone of Mauritius island was amplified with its corresponding specific sequevar host (I-15, I-31, I-33, see Supplementary Table 1 for details). High titers were produced by infecting 25 mL of the host strains with 0.1 mL of each phage isolate. Following a standard chloroform protocol phages lysates were produced and kept at 4 °C until needed^21^.

### Bacterial strains and culture media

All *Ralstonia solanacearum* species complex strains used here belong to the reference collection of the Pôle de Protection des Plantes in Reunion Island (France). Details about their phylogenetic classification and origin are provided in Supplementary Table 2. All inoculations and bacterial cultures were carried out in a semi-selective Kelman culture medium containing 1% m/m triphenyltetrazolium chloride. All bacterial cultures were incubated at 28 °C. Liquid cultures were agitated at 80 rpm when they included phages or 120 rpm for bacteria only.

### Phage genome extraction and sequencing

Genomic DNA extraction of 81 phages from a high titer phage lysate was done with the Wizard DNA clean-up kit from Promega. Restriction analysis of phage genomic DNA was performed with the enzymes NcoI, PstI and HindIII (Promega), after failed DNA digestion with EcoRI, XbaI et KnpI, and thus phages classified by restriction profile group. One phage per group was selected for whole genome sequencing. These results and the genomic DNA quality guided the selection of 39 phages for whole genome sequencing. DNA phage libraries were prepared with the TruSeq Nano kit (Illumina) for 39 phage genomes and sequenced to 2 × 250 pb reads length using Illumina HiSeq at the sequencing platform GeT-PlaGe of GenoToul (Toulouse, France).

### Phage genome assembly and annotation

Reads were trimmed with Sickle 1.33.2 and assembled with SPAdes 3.12.0^22,23^. Coding sequences (CDSs) were predicted and annotated using Prokka 1.14.5, and manual refinement database searches were performed using BLASTp, HHpred and VIRFam to predict head-neck-tail module genes^24–27^.

### Phage life-style prediction

Temperate phages were identified as in other works^28^. Briefly all phage genomes were analysed using PHACTS, PHASTER^29,30^. When the results were not conclusive we did a blast of the aminoacid sequences for the presence of recombinases using PFAM v26 and HMMER3 with default options^31,32^. These predictions were manually curated.

### Phage phylogenetic analysis

Phylogenetic tree based on nucleotides were constructed using VICTOR^17^. The branch lengths were scaled in terms of the Genome BLAST Distance Phylogeny (GBDP) intergenomic distance formula d0 for nucleotide based VICTOR phylogenetic trees^17^. A proteomic tree was constructed using the ViPTree server based on genome-wide sequence similarities computed by tBLASTx^33^.

### TEM micrographs

Electron micrographs of *R. solanacearum* phages were generated as in other studies^34^. Briefly, the specimen was picked up with 400-mesh copper grids (Delta microscopies, Mauressac, French), negative stained with 1% (W/V) ammonium molybdate, and analysed using a HT7800 Hitachi transmission electron microscope (Hitachi, Tokyo, Japan) at an acceleration voltage of 80 kV. Electron micrographs were taken with an AMT XR401, sCMOS-camera (AMT imaging, Woburn, MA-US).

## Results and discussion

### Sampling and geographical distribution of phages

**Figure 1:**
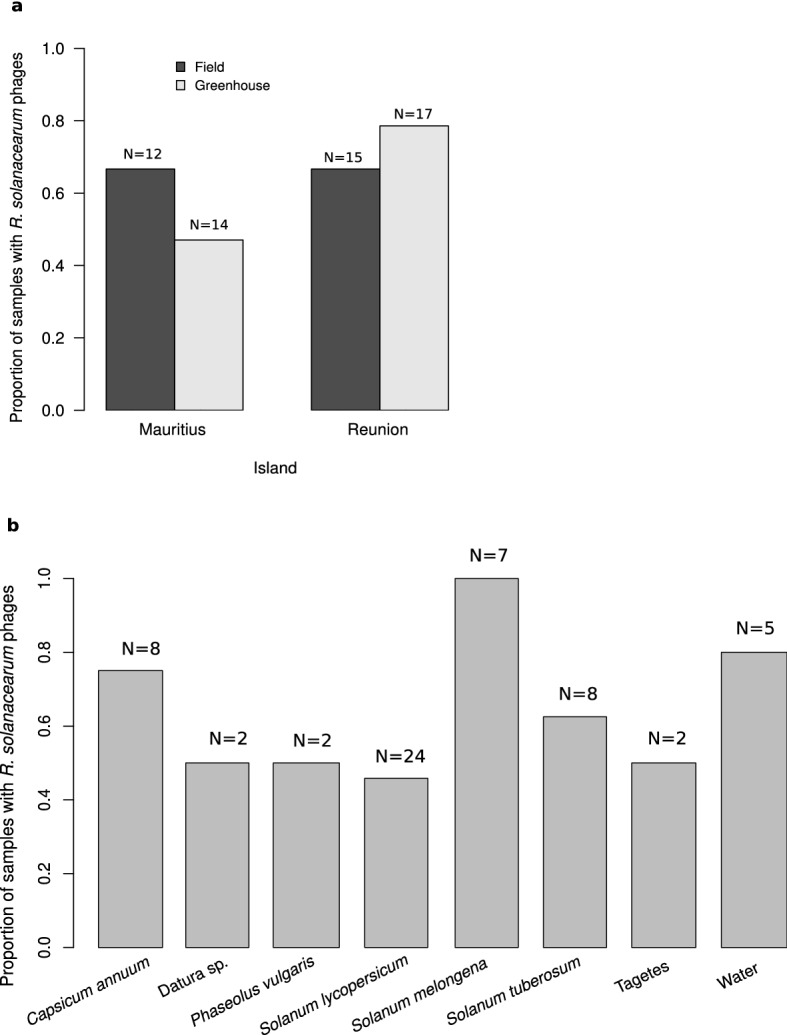
Proportion of samples positive for *R. solanacearum* phages, by island of origin and agricultural practices (**a**), and host plant or type of sample (**b**).

*R. solanacearum* phages were broadly present in agricultural material associated with bacterial wilt from Reunion (76%, N = 26), and Mauritius (56%, N = 32) (Fig. 1a, Supplementary Fig. 1, Supplementary Table 2). There were no significant differences in the proportion of positive samples between the two Southwest Indian Ocean islands (quasi-Poisson GLM: F = 1.887, d.f. = 1, p-value = 0.303, Fig. 1a). Samples came from greenhouses or fields, with no differences between these agricultural settings in terms of phage abundance (quasi-Poisson GLM: F = 0.06, d.f. = 1, p-value = 0.830, Fig. 1a). *R. solanacearum* phages were found in around 50% or more of all types of samples, from plants, soil from uprooted fields and water (Fig. 1b). The highest proportion of positive samples were observed in aubergine (*Solanum melongena*), pepper (*Capsicum annum*), and irrigation water samples (Fig. 1b).

Not all bacterial strains were susceptible to the phages, as revealed by the subset of RSSC strains that were attacked by the phages in the enrichment step of the phage isolation (Supplementary Table 1). Only strains from phylotype I resulted in the isolation of phages, a result likely derived from the epidemiology of this bacterium in the islands, where this is the main RSSC genetic type present, and the specificity and local adaptation of phages^3^. After purification and differentiation of individual lysis plaques, a total of 35 and 46 individual phages were isolated from Reunion and Mauritius, but only 17 and 21 phages were sequenced, respectively, after discarding identical DNA digestion profiles through analysis with different restriction enzymes. Sequencing allowed the identification of ten different phage genomes from Reunion and 13 from Mauritius, and the rest were identical clones (Table 1). We named Mauritius phages after historical cyclones that hit the island, and Reunion ones after legendary anti-colonialist slaves. We included a gender balance criteria in the choice of phage names. The most abundant phage clones were Ralstonia phage Bakoly (*Ralstonia virus Bakoly*) found five times in Mauritius, and Ralstonia phage Cimandef (*Ralstonia virus Cimandef*) found three times in Reunion, in different host plants (Supplementary Table 1).

**Table 1:** Taxonomic and genomic features of 23 newly isolated *R. solanacearum* phages.

Family	Genus	Species	Phage	Genome size (bp)	GC content (%)	Protein number	Coverage	GenBank accession numbers	Origin
*Autographiviridae*	*Anchaingvirus*	*Ralstonia virus Anchaing*	Anchaing	43,597	63.09	53	153	MT740728	Reunion
*Myoviridae*	*Bakolyvirus*	*Ralstonia virus Bakoly*	Bakoly	44,222	62.32	62	181	MT740729	Mauritius
			Jenny	43,921	62.31	62	236	MT740744	Mauritius
		*Ralstonia virus Simangalove*	Adzire	44,732	62.23	58	251	MT740725	Reunion
			Elie	44,966	62.33	58	196	MT740735	Reunion
			Sarlave	44,858	62.26	58	255	MT740746	Reunion
			Simangalove	44,834	62.25	58	211	MT740747	Reunion
*Podoviridae*	*Cimandefvirus*	*Ralstonia virus Cimandef*	Cimandef	55,171	64.83	66	165	MT740730	Reunion
			Dimitile	57,009	64.58	65	126	MT740733	Reunion
		*Ralstonia virus Heva*	Heva	58,352	64.62	69	114	MT740742	Reunion
		*Ralstonia virus Eline*	Eline	57,352	64.62	66	105	MT740736	Mauritius
		*Ralstonia virus Gamede*	Gamede	58,603	64.60	68	91	MT740738	Mauritius
		*Ralstonia virus Gerry*	Gerry	60,898	64.73	78	118	MT740739	Mauritius
*Siphoviridae*	*Dinavirus*	*Ralstonia virus Dina*	Dina	39,161	65.27	55	68	MT740734	Mauritius
*Podoviridae*	*Firingavirus*	*Ralstonia virus Firinga*	Firinga	39,744	59.69	51	239	MT740737	Mauritius
			Hennie	39,476	59.44	54	10	MT740741	Mauritius
*Podoviridae*	*Gervaisevirus*	*Ralstonia virus Claudettte*	Alix	57,681	64.32	71	102	MT740727	Mauritius
			Claudette	57,085	64.37	71	80	MT740731	Mauritius
		*Ralstonia virus Gervaise*	Darius	60,734	64.83	74	41	MT740732	Mauritius
			Gervaise	61,164	64.72	73	145	MT740740	Mauritius
*Myoviridae*	*Rahariannevirus*	*Ralstonia virus Raharianne*	Albius	46,541	60.62	75	181	MT740726	Reunion
			Hyacinthe	46,075	60.50	75	192	MT740743	Mauritius
			Raharianne	46,321	60.55	75	157	MT740745	Reunion

Italics lines indicate phage clones from Reunion and white ones phages from Mauritius.

As it will be detailed in the next section, the 23 phages of the study represent 13 new species, within seven new genera which belong to four different phage families. Three genera out of the seven described were found in both islands, one was so far endemic of Reunion and one of Mauritius, and two contained Mauritian and foreign phages. Five different phage species were distinguished in Reunion, whereas eight different ones were found in Mauritius. Most phage species were geographically restricted to one of the islands, except *Ralstonia virus Raharianne*, that was found in both Mauritius and Reunion. Even for this shared phage, *Ralstonia virus Raharianne*, clones were unique from each island, with two representatives in Reunion (Ralstonia phage Raharianne and Ralstonia phage Albius) and one in Mauritius (Ralstonia phage Hyacinthe).

The sampling design of this study was not exhaustive enough to present general conclusions on the geographical distribution of *R. solanacearum* phages in the islands, or the association with specific host plants and agricultural practices, but the results support the presence of *R. solanacearum* phages in all agricultural environments where bacterial wilt was recorded. We provide an important collection of *R. solanacearum* phages that considerably widens its known genetic diversity. Mauritius phages are more diverse, with more genera and species, compared to Reunion ones in this collection, a non surprising fact, in light of the more complex and spatially structured RSSC epidemiology in Mauritius^3^. All clones are only located in one of the islands, suggesting an evolutionary process of local adaptation, although some genera are shared, indicating a phylogenetic relationship of *R. solanacearum* phages within the Southwest Indian Ocean, likely linked to the related bacterial epidemiology^3^.

### Taxonomic analysis and support for the description of seven new *R. solanacearum* phage genera

The 23 phages of our study represent 13 new species, within seven new genera, according to the scored phylogenomic distances calculated using VIRIDIC (Virus Intergenomic Distance Calculator), which integrates the ICTV criteria (Table 2). Four genera (*Anchaingvirus*, *Dinavirus*, *Firingavirus* and *Rahariannevirus*) include only one phage species. The genera *Bakolyvirus* and *Gervaisevirus* include two new *R. solanacearum* phage species each, and five new phage species belong to the *Cimandefvirus* genus, being the most diverse one of the seven (Table 2). The BLASTn analysis of the 23 phages isolated in this study did not reveal any significant genetic similarities to other phages in databases (last accessed in July 2020) at the nucleotide level (< 75%, ICTV criterion), except for 3 *R. solanacearum* phages sampled in China and Japan that were relatively similar to ours: Ralstonia phage RPZH6, Ralstonia phage GP4 and Ralstonia phage RSK1, (Supplementary Table 3)^35^. These three phages have not been completely taxonomically classified and, according to the nucleotide similarity with the presently studied phages, we suggest to include them in the new genera *Gervaisevirus* (RPZH6 and GP4) and *Firingavirus* (RSK1).

**Table 2:**
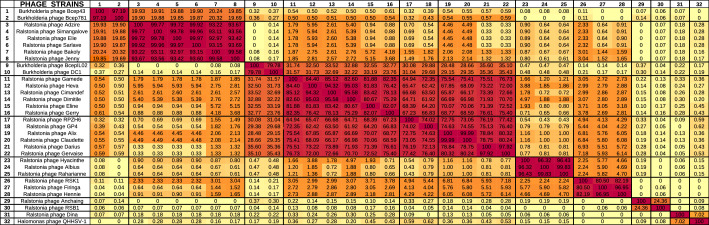
The VIRIDIC pairwise intergenomic distances/similarities amongst the 23 phage genomes isolated in our study and the nine closest phages as determined by BLASTN analysis.

Phages belonging to the same genera are differentiated by black lines, with sequence homology over 70% and red colour code in the heatmap. Sequence homology over 95% and the darker red colour code is associated with the species taxon level.

**Figure 2:**
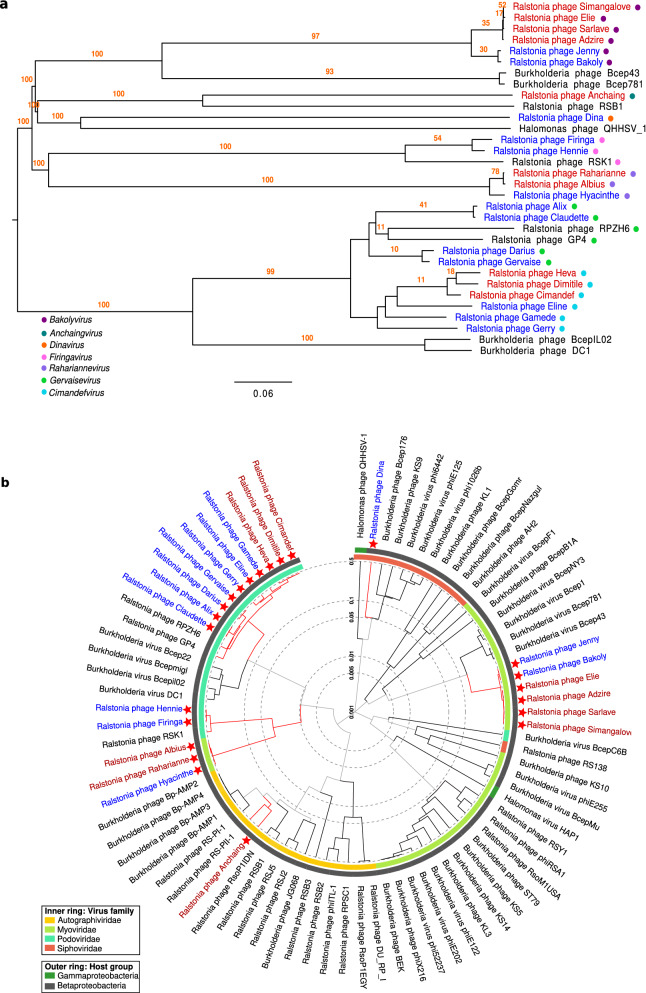
Phylogenetic trees based on the full genome of the new 23 *R. solanacearum* phages (in red those from Reunion island and in blue the ones from Mauritius). (**a**) Tree based in nucleotides including also the nine closest phage relatives (in black), calculated with VICTOR methods. The seven proposed genera are indicated by coloured dots on the left side of the tree. A bootstrap value of 100 was used in the analysis. (**b**) Circular proteomic tree generated by ViPTree including also all *R. solanacearum* (non-Inoviridae and non-jumbo), *Burkholderia* and *Halomonas* phages, coloured by virus families and host taxonomic groups. The new 23 *R. solanacearum* phages are indicated by red stars.

Phylogenetic trees using the full genome of the new 23 phages and including nine closest relatives provide additional support to the genera classification by VIRIDIC, with highly consistent branches (higher or equal to 96 bootstrap replicates) (Fig. 2a). The closest viruses, included in this tree, are four *R. solanacearum* phages, four *Burkholderia* phages and one *Halomonas* phage isolated from China, Japan and the USA^35–38^. Taxonomically, *Burkholderia* and *Ralstonia* bacteria are from different genus (*Ralstonia* and *Burkholderia*), but the same family (*Burkholderiaceae*), and thus the distant relationship of phages that attack them is not unexpected. However, *Halomonas* bacteria are classified as Gammaproteobacteria, a different bacterial class than the one assessed to *Ralstonia* (Betaproteobacteria). Yet, the genetic homology between the related *Halomonas* phage and our *R. solanacearum* phages is only 4% (Supplementary Table 3). Just two complete genomes of *Halomonas* phages have been described so far, according to GenBank records. The proteomic tree generated with ViPTree confirmed the taxonomic relationships among the *R. solanacearum* phages of the present study, as well as their genomic uniqueness compared to previously described *R. solanacearum*, *Burkholderia* and *Halomonas* phages (Fig. 2b). This may be explained by the geographical distance of the Southwest Indian Ocean islands from previous phage samples, the genetic diversity of the RSSC and overall biodiversity present. The high diversity and quantity of new phage genera and species described in our study is all the same surprising.

**Figure 3:**
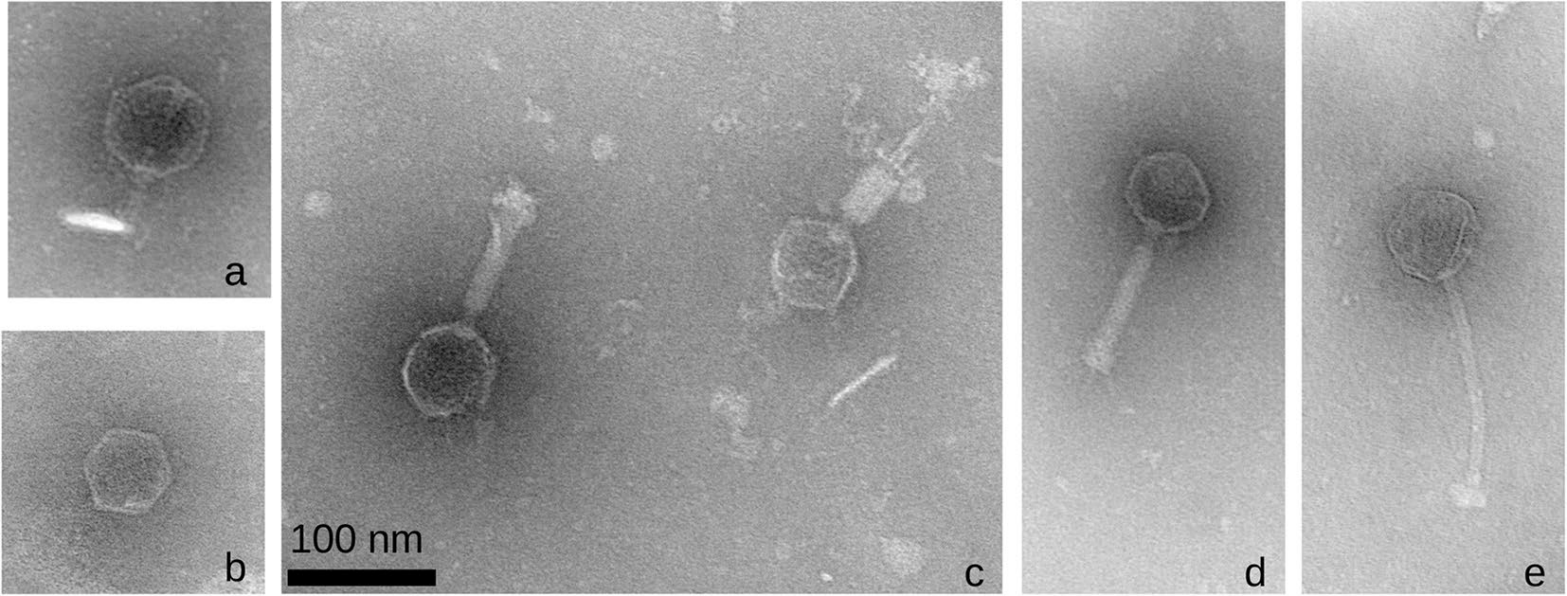
Transmission electron micrographs of the representative *R. solanacearum* phage species of five new genera with unique viruses (non described before): (**a**) Ralstonia phage Cimandef (*Cimandefvirus, Podoviridae*); (**b**) Ralstonia phage Anchaing (*Anchaingvirus, Autographiviridae*); (**c**) Ralstonia phage Raharianne (*Rahariannevirus, Myoviridae*); (**d**) Ralstonia phage Bakoly (*Bakolyvirus, Myoviridae*); (**e**) Ralstonia phage Dina (*Dinavirus, Siphoviridae*).

The electron micrographs confirm the Virfam genetic-based predictions of phage families in this phage collection, that is, the seven genera are classified as four *Podoviridae*, one *Siphoviridae* and two *Myoviridae*^25^ (Fig. 3). The presence of RNA and DNA polymerases in the genome of *Ralstonia virus Anchaing* revealed that this *Podoviridae* is in fact an *Autographiviridae*^39^. Surprisingly, only one *Podoviridae* and one *Siphoviridae* had been described before amongst the previous *R. solanacearum* phage species. In the present *R. solanacearum* phage collection we assess nine species as new *Podoviridae*, one new *Siphoviridae* and one new *Autographiviridae R. solanacearum* phage (Table 1, Fig. 3).

### Genomic comparison of the seven new genera

The average genome size of the 23 Southwest Indian Ocean phages was 50,108 bp, the average GC content 63%, and the average number of encoded proteins was 65. Compared to the genome size of 22 other non-*Inoviridae* and non-jumbo (giant) *R. solanacearum* phages already described (41,536 bp), the present phage collection average genome size is bigger. The biggest genome size in the present collection was the 61,164 bp of Ralstonia phage Gervaise, and the smallest genome was that of Ralstonia phage Dina, with 39,161 bp. This genus, *Dinavirus* had also the highest GC content (65.27%). This is consistent with previous analysis that correlate GC % with low genome size in phages^40^. It was not the case of the two phage species of the genus *Firingavirus*, with small genome size (39,610 bp) and the lowest GC % (59.56) of the entire collection. As seen for other phages, the average GC% of all 23 phages is lower than that of the bacterial host, which has a median GC% of 66.8^40^. As for the number of proteins encoded, there was variability within the new phage genera, and overall it ranged from the 51 of Ralstonia phage Firinga to the 78 of Ralstonia phage Gerry.

The ability of phages to perform lysogenic life-cycles (temperate phages) is not always elucidated or discussed in genomic or phenotypic studies. From the seven *R. solanacearum* phage genera studied here, three of them are probably composed by temperate phages: *Dinavirus*, *Anchaingvirus* and *Gervaisevirus*, as suggested by the presence of a gene encoding a recombinase in their genome. The lack of experimental proof of the integrase activity in these or the homologous genes in current databases, precludes more solid conclusions. Phages from the other four genera are with high probability virulent viruses, thus only capable of performing lytic cycles. This implies a significant (26%) proportion of *R. solanacearum* phages potentially able to undergo a lysogenic cycle, but a higher proportion of mostly virulent phages and therefore adapted for biocontrol purposes. Interestingly, from the four temperate phage species, three were sampled in Mauritius and only one in Reunion island (*Ralstonia virus Anchaing*). One potential cause of the abundance of temperate phages in Mauritius is the tight molecular interactions and genetic exchanges with their hosts, related to the more complex RSSC epidemiology in this island^3^. In Reunion, a majority of virulent phages may have faster effects for the more homogeneous RSSC bacterial populations. Further surveys and experiments would be needed to test these ideas.

**Figure 4:**
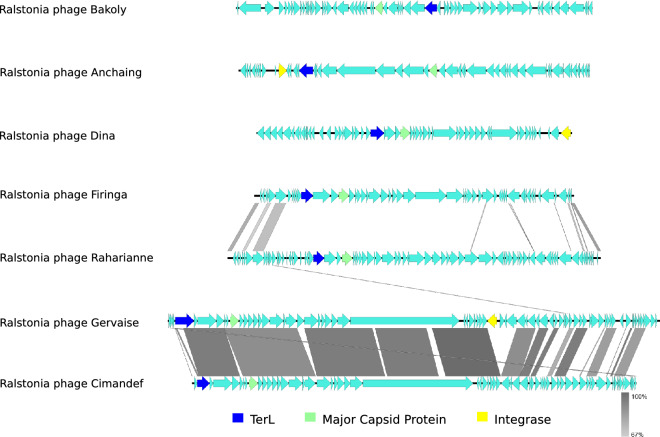
Synteny plot of the reference phages of each of the seven new *R. solanacearum* virus genera. Coding regions are represented by cyan arrows, nucleotide homology (BlastN) in grey and conserved proteins are indicated in colour codes. Figure was generated with EasyFig.

Finally, substantial genomic homology can be observed between the reference phages of the genera *Cimandefvirus* and *Gervaisevirus*, really weak between *Gervaisevirus*, *Rahariannevirus* and *Firingavirus*, and completely absent for *Dinavirus*, *Anchaingvirus* and *Bakolyvirus* (Fig. 4). The absence of synteny provides further evidence of the non-conserved genomic architecture between this particular group of *R. solanacearum* phages (Fig. 4).

## Conclusions

There is a substantial imbalance in virtually all host–parasite interactions, with multiple parasites attacking the same host species. This is the case for bacteria and phages, but the knowledge and amount of isolated or sequenced bacteria surpass that of their natural enemies, bacteriophages^41^. This study reports a wide diversity of newly isolated *R. solanacearum* phages in two islands of the Southwest Indian Ocean, and demonstrates the prevalence of the parasites of this bacterium in agricultural environments affected by bacterial wilt disease. Only 35 phages of *R. solanacearum* have been previously fully analysed at the genomic level. Earlier phages were mostly sampled in eastern Asiatic countries, and other geographic origins have been largely disregarded. The 20 non-Inoviridae *R. solanacearum* phages previously known are classified into fourteen genera, revealing a high taxonomic variety. Genetic differences between our phages and other fully-sequenced *R. solanacearum* phages reported in current databases are substantial, in most cases characterized by the complete absence of genetic homology. We identify nonetheless, three phages belonging to the newly defined genera. Following the ICTV guidelines, we propose seven new genera of *R. solanacearum* phages, which include twenty-three new phage clones within thirteen new species. A small proportion of the isolated phages are probably temperate, and specially abundant in Mauritius. In this island, phage genetic diversity is significantly higher than in Reunion. Taken together, these results suggest different phage-bacteria dynamics and evolutionary history of *R. solanacearum* phages in each island, which deserve additional investigations.

This work is singular for many reasons: the quantity and diversity of new *R. solanacearum* phages presented, their unprecedented geographical origin, the complete disclosure of the sampling results (including negative results, nature of the samples and exact location), and the deep genomic analysis characterisation. Our work expands substantially the existing *R. solanacearum* phage records and points in the direction that there is much more phage genetic diversity waiting to be discovered in agricultural ecosystems. Furthermore, broad comprehension of the genetic features of phages and their association with their bacterial hosts’ local epidemiology is essential to fine-tune biocontrol strategies based on phages against bacterial pathogens.

## Supplementary Information

Supplementary Information.

## Data Availability

Assembled and annotated genomes of the 23 *R. solanacearum* phages were uploaded to GenBank under accession numbers MT740725 to MT740747. All data generated or analysed during this study are included in this published article (and its “Supplementary Information files”).
