# Supplementary material for: High genomic diversity of novel phages infecting the plant pathogen Ralstonia solanacearum, isolated in Mauritius and Reunion islands
Source: Sci Rep. 2021 Mar 8;11:5382. doi: 10.1038/s41598-021-84305-7 (PMC7940629; doi:10.1038/s41598-021-84305-7)
Supplement: Supplementary file 1 — Supplementary Information. [file 41598_2021_84305_MOESM1_ESM.pdf]

High genomic diversity of novel phages infecting the plant pathogen *Ralstonia solanacearum*, isolated in Mauritius and Reunion islands

Angelina Trotereau[1], Claudine Boyer[2], Isabelle Bornard[3], Max Jean Bernard Pécheur[4], Catherine Schouler[1], Clara Torres-Barceló[2,3]*

1 INRAE, ISP, Université de Tours, F-37380, Nouzilly, France
2 Plant Populations and Bio-aggressors in Tropical Ecosystems, Saint Pierre, Reunion, France.
3 INRAE, Plant Pathology, F-84140, Montfavet, France
4 Plant Pathology Division, FAREI, Reduit, Mauritius
* email: clara.torresbarcelo@inrae.fr

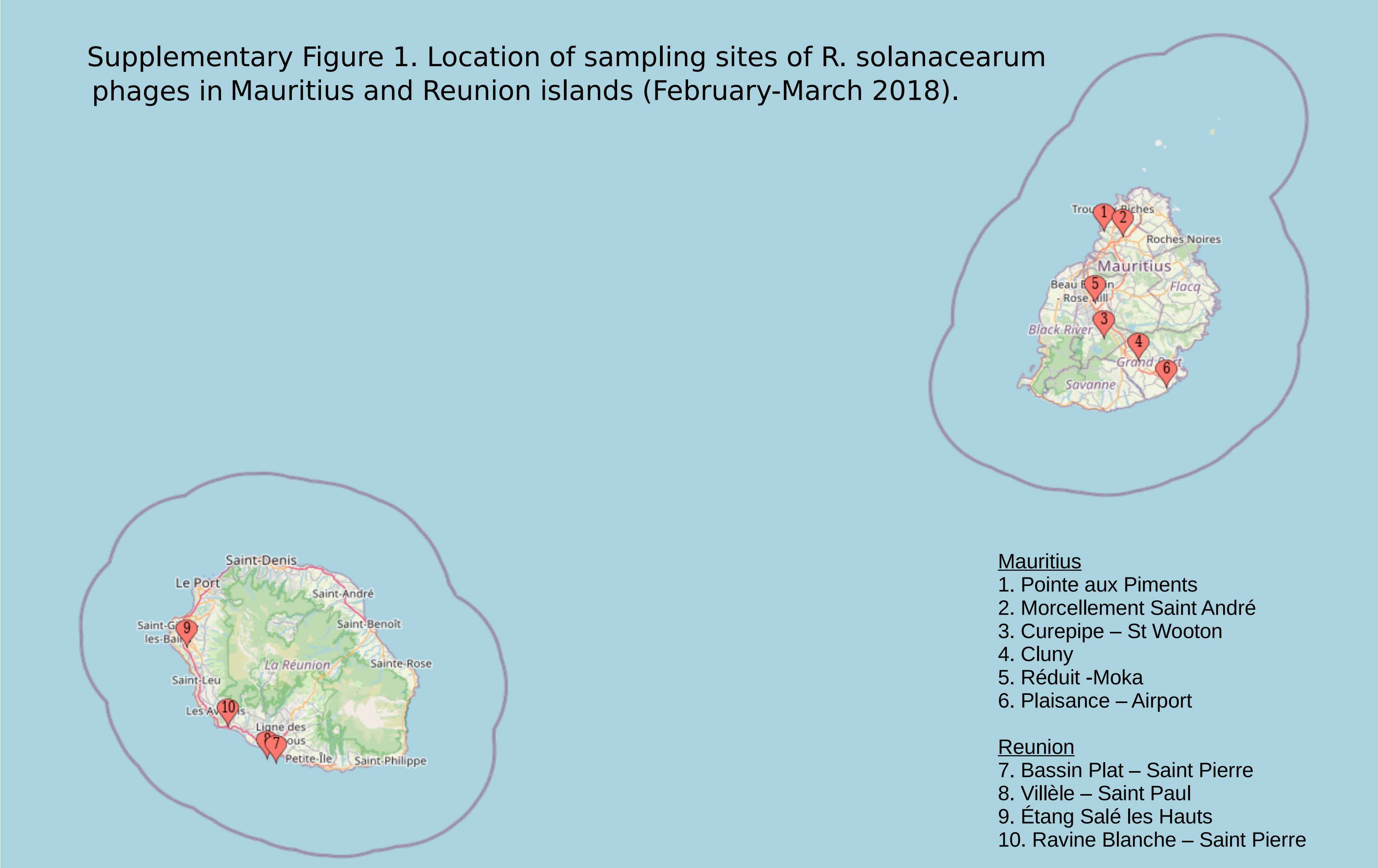

Supplementary Figure 1. Location of sampling sites of R. solanacearum phages in Mauritius and Reunion islands (February-March 2018).

<u>Mauritius</u>

1. Pointe aux Piments
2. Morcellement Saint André
3. Curepipe – St Wooton
4. Cluny
5. Réduit -Moka
6. Plaisance – Airport

<u>Reunion</u>

7. Bassin Plat – Saint Pierre
8. Villèle – Saint Paul
9. Étang Salé les Hauts
10. Ravine Blanche – Saint Pierre

Map created with: Mapcustomizer.com

Supplementary Table 1. R. solanacearum phage sampling information carried out in Mauritius and Reunion islands (February-March 2018).

Island	Sample	Localization	Plant or type of sample	Agricultural condition	Phage presence	Bacterial strain sequevar	Bacterial strain code	Isolated phage
Mauritius	1	Pointe aux Piments	*Solanum melongena*	Field	yes	I-15	RUN4382	nd
Mauritius	1	Pointe aux Piments	*Solanum melongena*	Field	yes	I-15	RUN4407	Gervaise
Mauritius	1	Pointe aux Piments	*Solanum melongena*	Field	yes	I-15	RUN4407	Gervaise
Mauritius	2	Pointe aux Piments	*Solanum melongena*	Field	yes	I-15	RUN4382	Darius
Mauritius	2	Pointe aux Piments	*Solanum melongena*	Field	yes	I-33	RUN4396	Bakoly
Mauritius	2	Pointe aux Piments	*Solanum melongena*	Field	yes	I-15	RUN4407	Dina
Mauritius	2	Pointe aux Piments	*Solanum melongena*	Field	yes	I-15	RUN4407	nd
Mauritius	2	Pointe aux Piments	*Solanum melongena*	Field	yes	I-33	RUN4680	Bakoly
Mauritius	2	Pointe aux Piments	*Solanum melongena*	Field	yes	I-33	RUN4833	nd
Mauritius	3	Pointe aux Piments	*Capsicum annuum*	Field	yes	I-33	RUN4396	Bakoly
Mauritius	3	Pointe aux Piments	*Capsicum annuum*	Field	yes	I-15	RUN4407	nd
Mauritius	3	Pointe aux Piments	*Capsicum annuum*	Field	yes	I-33	RUN4680	nd
Mauritius	3	Pointe aux Piments	*Capsicum annuum*	Field	yes	I-33	RUN4833	nd
Mauritius	3	Pointe aux Piments	*Capsicum annuum*	Field	yes	I-33	RUN4833	Eline
Mauritius	4	Pointe aux Piments	*Capsicum annuum*	Field	yes	I-15	RUN4382	nd
Mauritius	5	Pointe aux Piments	*Capsicum annuum*	Field	yes	I-33	RUN4396	Bakoly
Mauritius	6	Pointe aux Piments	*Capsicum annuum*	Field	yes	I-33	RUN4396	nd
Mauritius	7	Morcellement Saint André	*Solanum melongena*	Field	yes	I-15	RUN4407	Gervaise
Mauritius	7	Morcellement Saint André	*Solanum melongena*	Field	yes	I-15	RUN4407	nd
Mauritius	8	Morcellement Saint André	*Solanum melongena*	Field	yes	I-31	RUN5163	Hyacinthe
Mauritius	9	Morcellement Saint André	*Phaseolus vulgaris*	Field	yes	I-31	RUN5163	Gamede
Mauritius	9	Morcellement Saint André	*Phaseolus vulgaris*	Field	yes	I-31	RUN5163	nd
Mauritius	10	Morcellement Saint André	*Phaseolus vulgaris*	Field	no	na	na	na
Mauritius	11	Curepipe – St Wooton	*Tagetes*	Greenhouse	no	na	na	na
Mauritius	12	Curepipe – St Wooton	*Tagetes*	Greenhouse	yes	I-15	RUN4407	Hennie
Mauritius	13	Cluny	*Solanum lycopersicum*	Greenhouse	no	na	na	na
Mauritius	14	Cluny	*Solanum lycopersicum*	Greenhouse	no	na	na	na
Mauritius	15	Cluny	*Solanum lycopersicum*	Greenhouse	no	na	na	na
Mauritius	16	Cluny	*Solanum lycopersicum*	Greenhouse	no	na	na	na
Mauritius	17	Cluny	*Solanum lycopersicum*	Greenhouse	no	na	na	na
Mauritius	18	Cluny	*Solanum lycopersicum*	Greenhouse	yes	I-15	RUN4407	Firinga
Mauritius	19	Cluny	*Solanum lycopersicum*	Greenhouse	no	na	na	na
Mauritius	20	Cluny	*Solanum lycopersicum*	Greenhouse	yes	I-15	RUN4382	Alix
Mauritius	20	Cluny	*Solanum lycopersicum*	Greenhouse	yes	I-15	RUN4407	Claudette
Mauritius	20	Cluny	*Solanum lycopersicum*	Greenhouse	yes	I-15	RUN4407	nd
Mauritius	20	Cluny	*Solanum lycopersicum*	Greenhouse	yes	I-33	RUN4833	nd
Mauritius	21	Cluny	*Solanum lycopersicum*	Greenhouse	no	na	na	na
Mauritius	22	Cluny	*Solanum lycopersicum*	Greenhouse	yes	I-15	RUN4382	Claudette
Mauritius	22	Cluny	*Solanum lycopersicum*	Greenhouse	yes	I-15	RUN4407	nd
Mauritius	22	Cluny	*Solanum lycopersicum*	Greenhouse	yes	I-15	RUN4407	nd
Mauritius	22	Cluny	*Solanum lycopersicum*	Greenhouse	yes	I-33	RUN4833	nd
Mauritius	22	Cluny	*Solanum lycopersicum*	Greenhouse	yes	I-31	RUN5163	nd
Mauritius	22	Cluny	*Solanum lycopersicum*	Greenhouse	yes	I-31	RUN5163	nd
Mauritius	23	Plaisance	*Solanum lycopersicum*	Greenhouse	yes	I-15	RUN4407	nd
Mauritius	23	Plaisance	*Solanum lycopersicum*	Greenhouse	yes	I-33	RUN4680	Bakoly
Mauritius	23	Plaisance	*Solanum lycopersicum*	Greenhouse	yes	I-33	RUN4833	Gerry
Mauritius	23	Plaisance	*Solanum lycopersicum*	Greenhouse	yes	I-31	RUN5163	nd
Mauritius	24	Plaisance	*Solanum lycopersicum*	Greenhouse	yes	I-33	RUN4833	nd
Mauritius	24	Plaisance	*Solanum lycopersicum*	Greenhouse	yes	I-33	RUN4833	nd

Mauritius	24	Plaisance	*Solanum lycopersicum*	Greenhouse	yes	I-31	RUN5163	nd
Mauritius	25	Reduit	*Capsicum annuum*	Field	no	na	na	na
Mauritius	26	Reduit	*Capsicum annuum*	Field	no	na	na	na
Mauritius	27	Pointe aux Piments	Water tank	Field	yes	I-33	RUN4833	nd
Mauritius	28	Cluny	Water tank	Serre	no	na	na	na
Mauritius	29	Plaisance	Water tank after UV treatment	Greenhouse	yes	I-31	RUN5163	nd
Mauritius	30	Plaisance	Water tank before UV treatment	Greenhouse	yes	I-33	RUN4396	Jenny
Mauritius	30	Plaisance	Water tank before UV treatment	Greenhouse	yes	I-33	RUN4680	nd
Mauritius	30	Plaisance	Water tank before UV treatment	Greenhouse	yes	I-33	RUN4833	nd
Mauritius	30	Plaisance	Water tank before UV treatment	Greenhouse	yes	I-33	RUN4833	Bakoly
Mauritius	31	Reduit	Soil old *Solanum lycopersicum* field	Field	no	na	na	na
Mauritius	32	Reduit	Soil old *Solanum lycopersicum* field	Field	no	na	na	na
Reunion	1	Bassin Plat – Saint Pierre	*Solanum lycopersicum*	Greenhouse	yes	I-31	RUN3014/RUN3665	Anchaing
Reunion	2	Bassin Plat – Saint Pierre	*Solanum lycopersicum*	Greenhouse	yes	I-31	RUN3014/RUN3665	Cimandef
Reunion	3	Bassin Plat – Saint Pierre	*Solanum lycopersicum*	Greenhouse	yes	I-31	RUN3014/RUN3665	Cimandef
Reunion	3	Bassin Plat – Saint Pierre	*Solanum lycopersicum*	Greenhouse	yes	I-31	RUN3014/RUN3665	Cimandef
Reunion	4	Bassin Plat – Saint Pierre	*Solanum melongena*	Field	yes	I-31	RUN3014/RUN3665	Dimitile
Reunion	4	Bassin Plat – Saint Pierre	*Solanum melongena*	Field	yes	I-31	RUN3014/RUN3665	Dimitile
Reunion	5	Bassin Plat – Saint Pierre	Soil	Field	yes	I-31	RUN3014/RUN3665	Cimandef
Reunion	5	Bassin Plat – Saint Pierre	Soil	Field	yes	I-31	RUN3014/RUN3665	Cimandef
Reunion	5	Bassin Plat – Saint Pierre	Soil	Field	yes	I-31	RUN3014/RUN3665	Cimandef
Reunion	6	Villèle – Saint Paul	Soil from old *Solanum tuberosum* field	Champs	yes	I-31	RUN3014/RUN3665	Heva
Reunion	7	Villèle – Saint Paul	Soil from old *Solanum tuberosum* field	Champs	no	na	na	na
Reunion	8	Villèle – Saint Paul	Soil from old *Solanum tuberosum* field	Champs	yes	I-31	RUN3014/RUN3665	nd
Reunion	9	Villèle – Saint Paul	*Solanum tuberosum*	Champs	yes	I-31	RUN3014/RUN3665	nd
Reunion	10	Villèle – Saint Paul	*Solanum tuberosum*	Champs	yes	I-31	RUN3014/RUN3665	nd
Reunion	11	Villèle – Saint Paul	*Solanum tuberosum*	Champs	no	na	na	na
Reunion	12	Villèle – Saint Paul	*Solanum tuberosum*	Champs	no	na	na	na
Reunion	13	Villèle – Saint Paul	*Solanum tuberosum*	Champs	yes	I-31	RUN3014/RUN3665	nd
Reunion	14	Etang Salé les Hauts	*Solanum lycopersicum*	Greenhouse	no	na	na	na
Reunion	15	Etang Salé les Hauts	*Solanum lycopersicum*	Greenhouse	no	na	na	na
Reunion	16	Etang Salé les Hauts	Water tank	Greenhouse	yes	I-31	RUN3014/RUN3665	Simangalove
Reunion	17	Etang Salé les Hauts	Soil outside greenhouse	Greenhouse	yes	I-31	RUN3014/RUN3665	Adzire
Reunion	17	Etang Salé les Hauts	Soil outside greenhouse	Greenhouse	yes	I-31	RUN3014/RUN3665	Sarlave
Reunion	18	Etang Salé les Hauts	Soil outside greenhouse	Greenhouse	yes	I-31	RUN3014/RUN3665	Elie
Reunion	19	Ravine Blanche – Saint Pierre	*Datura sp.*	Field	yes	I-31	RUN3014/RUN3665	Albius
Reunion	20	Ravine Blanche – Saint Pierre	*Datura sp.*	Field	no	na	na	na
Reunion	21	Ravine Blanche – Saint Pierre	*C. annum*	Greenhouse	yes	I-31	RUN3014/RUN3665	nd
Reunion	22	Ravine Blanche – Saint Pierre	*C. annum*	Greenhouse	yes	I-31	RUN3014/RUN3665	nd
Reunion	23	Ravine Blanche – Saint Pierre	*Solanum lycopersicum*	Greenhouse	yes	I-31	RUN3014/RUN3665	Raharianne
Reunion	24	Ravine Blanche – Saint Pierre	*Solanum melongena*	Greenhouse	yes	I-31	RUN3014/RUN3665	Raharianne
Reunion	25	Ravine Blanche – Saint Pierre	*Solanum melongena*	Greenhouse	yes	I-31	RUN3014/RUN3665	nd
Reunion	26	Ravine Blanche – Saint Pierre	*Solanum lycopersicum*	Greenhouse	no	na	na	na

Supplementary Table 2. Table of Ralstonia solanacearum species complex bacterial strains used to isolate-enrich R. solanacearum phages sampled. The origin (country and host plant) of each strain as well as their phylogenetic classification are detailed.

**Bacterial species**	**Phylotype**	**Sequevar**	**Code**	**Haplotype**	**Plant**	**Country**
R. pseudosolanacearum	I	I-15	RUN4382	MT014	*S. lycopersicum*	Mauritius
R. pseudosolanacearum	I	I-33	RUN4396	MT009	*S. lycopersicum*	Mauritius
R. pseudosolanacearum	I	I-15	RUN4407	MT017	*S. lycopersicum*	Mauritius
R. pseudosolanacearum	I	I-18	RUN4584	MT019	*S. tuberosum*	Mauritius
R. pseudosolanacearum	I	I-33	RUN4680	MT060	*S. tuberosum*	Mauritius
R. pseudosolanacearum	I	I-33	RUN4833	MT061	*S. tuberosum*	Mauritius
R. pseudosolanacearum	I	I-31	RUN5163	MT035	*S. tuberosum*	Mauritius
R. pseudosolanacearum	I	I-31	RUN3014	MT035	*S. melongena*	Reunion
R. pseudosolanacearum	I	I-14	RUN3570	MT010	*S. lycopersicum*	Reunion
R. pseudosolanacearum	I	I-31	RUN3665	MT043	*S. lycopersicum*	Reunion
R. solanacearum	II	IIA-36	RUN3812	nd	*S. lycopersicum*	Reunion
R. solanacearum	II	IIB-1	RUN35	nd	*S. tuberosum*	Netherlands
R. solanacearum	II	IIB-1	RUN4509	nd	*S. tuberosum*	Reunion
R. pseudosolanacearum	III	III-19	RUN3733	nd	*S. lycopersicum*	Reunion
R. pseudosolanacearum	III	III-29	RUN133	nd	*S. lycopersicum*	Cameroun
R. syzygii	IV	IV-10	RUN83	nd	*S. lycopersicum*	Indonesia
R. syzygii	IV	IV-10	RUN4606	nd	*S. tuberosum*	Mauritius

Supplementary Table 3. BLASTN homologous analysis phage genomes (last accessed in July 2020) to the reference phage species of the 7 Ralstonia phage new genera proposed. Phages with significant similarity at the nucleotide level (<75%) are highlighted in light yellow.

	Max Score	Total Score	Query Cover	E value	Per. Ident	Accession
**Ralstonia phage Gervaise; *Ralstonia virus Gervaise; Gervaisevirus***						MT740740
Ralstonia phage RPZH6, complete genome	12759	82628	78%	0	96.22%	MT361768.1
Ralstonia phage GP4, complete genome	12454	72696	78,00%	0	96,84%	MH638294.1
Burkholderia cenocepacia phage BcepIL02, complete genome	2963	8443	20,00%	0	77,78%	FJ937737.2
Burkholderia phage DC1, complete genome	2944	7684	20,00%	0	78,00%	JN662425.1
**Ralstonia phage Firinga; *Ralstonia virus Firinga; Firingavirus***						MT740737
Ralstonia phage RSK1 DNA, complete genome	45497	59186	79,00%	0	99,89%	AB863625.1
Ralstonia phage DU_RP_II, complete genome	2715	5550	9,00%	0	93,53%	MF150911.1
Ralstonia phage P4282 gene for bacteriolytic protein, complete cds	2403	2403	4,00%	0	91,96%	AB048798.1
Ralstonia phage RPZH6, complete genome	1057	3008	3,00%	0	94,71%	MT361768.1
**Ralstonia phage Cimandef; *Ralstonia virus Cimandef; Cimandefvirus***						MT740730
Ralstonia phage RPZH6, complete genome	10791	58511	73%	0	95.16%	MT361768.1
Ralstonia phage GP4, complete genome	10076	51925	69%	0	93.27%	MH638294.1
Burkholderia cenocepacia phage BcepIL02, complete genome	2527	8516	23%	0	80.81%	FJ937737.2
Burkholderia cepacia phage Bcep22, complete genome	2453	8626	23%	0	80.44%	AY349011.3
**Ralstonia phage Bakoly; *Ralstonia virus Bakoly; Bakolyvirus***						MT740729
Ralstonia phage GP4, complete genome	968	1145	1%	0	91.40%	MH638294.1
Ralstonia phage RsoP1IDN, complete genome	394	394	0%	4E-105	88.55%	NC_047930.1
Ralstonia phage RPZH6, complete genome	292	559	1%	2E-74	87.95%	MT361768.1
Ralstonia phage RSK1 DNA, complete genome	265	265	0%	4E-66	79.44%	AB863625.1
**Ralstonia phage Raharianne; *Ralstonia virus Raharianne; Rahariannevirus***						MT740745
Burkholderia phage DC1, complete genome	108	108	0%	7E-19	76.04%	JN662425.1
Ralstonia phage DU_RP_II, complete genome	71,3	71,3	0%	9E-08	93.62%	MF150911.1
Ralstonia phage RPZH6, complete genome	65,8	65,8	0%	4E-06	88.89%	MT361768.1
Ralstonia phage phiRSP, complete genome	65,8	65,8	0%	4E-06	93.18%	MH252365.1
**Ralstonia phage Anchaing; *Ralstonia virus Anchaing; Anchaingvirus***						MT740728
Ralstonia phage RS-PII-1, complete genome	2067	5234	21%	0	78.81%	NC_047804.1
Burkholderia phage AMP1, complete genome	1003	2528	12%	0	78.52%	MN191861.1
Burkholderia phage Bp-AMP4 complete genome	1003	2504	11%	0	78.52%	HG796221.1
Burkholderia phage Bp-AMP3 complete genome	1003	2528	12%	0	78.52%	HG796220.1
**Ralstonia phage Dina; *Ralstonia virus Dina; Dinavirus***						MT740734
Halomonas phage QHHSV-1, complete genome	680	680	4%	0	75.15%	KX879752.1
Mycobacterium phage MOOREtheMARYer, complete gen	52,8	52,8	0%	0,028	94.12%	NC_028791.1
